# Leisure time and occupational physical activity, overall and cardiovascular mortality: a 24-year follow-up in the OPERA study

**DOI:** 10.1080/07853890.2023.2245429

**Published:** 2023-08-16

**Authors:** Asla Suutari-Jääskö, Karri Parkkila, Juha Perkiömäki, Heikki Huikuri, Y. Antero Kesäniemi, Olavi H. Ukkola

**Affiliations:** Research Unit of Biomedicine and Internal Medicine, Medical Research Center Oulu, Oulu University Hospital, University of Oulu, Oulu, Finland

**Keywords:** Physical activity, Leisure time physical activity, occupational physical activity, mortality, cardiovascular mortality, heart failure

## Abstract

**Background:**

In earlier studies, the health benefits of physical activity have only been related to leisure time physical activity (LTPA). High occupational physical activity (OPA) might even be harmful. The current physical activity recommendations do not separate the OPA and LTPA. We investigated the effect of LTPA and OPA on cardiovascular morbidity and mortality during long-term follow-up. We also examined how heavy work affects the benefits of leisure time exercise.

**Material and methods:**

The study was part of the OPERA study and the baseline examinations were conducted between the years 1991 and 1993. The Follow-up of events continued until the end of the year 2020. Study subjects (*n* = 1044) were divided into four groups according to their LTPA (“no exercise”, “irregular”, “regular” and “heavy regular”) and into three groups according to their OPA (“no activity”, “mild” and “heavy”). The amount of exercise was self-reported and the exercise status was defined at the beginning of the study. Study subjects were followed up for their overall mortality (26 years), fatal and non-fatal CVD events (24 and 20 years) and heart failure (20 years). The survival analysis was performed using Kaplan–Meier curves and Cox-proportional hazard models.

**Results:**

“Heavy” OPA group subjects belonging to the “irregular” (less than 1–2 times 30 min exercise per week) LTPA group experienced the lowest overall mortality compared to other LTPA groups. Also, overall mortality was increased in the “mild” (*p* = 0.002) and CVD mortality in the” heavy” (*p* = 0.005) OPA group compared to “no activity”. The incidence of heart failure was increased in the “no exercise” LTPA compared to the “heavy regular” (*p* = 0.015) group.

**Conclusions:**

Study subjects who were in physically demanding occupations (heavy OPA) seemed to benefit from less LTPA than WHO currently recommends. Thus we suggest targeting different LTPA recommendations to different OPA groups.

## KEY MESSAGES

- Overall mortality was increased in the “mild” and CVD mortality in the” heavy” OPA group compared to “no activity” OPA in 26-year follow-up.
- Study subjects in physically demanding occupations benefitted more from less LTPA than the WHO currently recommends.
- High LTPA protected middle-aged study subjects from heart failure compared with sedentary study subjects at 20-year follow-up.

## 1. Introduction

The role of physical activity in lowering overall mortality and causing specific mortality and morbidities has been clear for decades [1]. However, more recently, several studies have reported that the health benefits of physical activity are exclusively related to leisure time physical activity (LTPA) [2–5]. Earlier studies have shown that a small amount of LTPA, even half of the WHO 2020 minimum physical activity criteria, showed significant benefits in overall health [6,7]. The WHO and Physical Activity Guidelines for Americans (2nd edition) recommend that everyone should exercise 150–300 min moderately per week or 75–150 min per week vigorously, or some equivalent amount of these combined. Current physical activity recommendations do not separate OPA and LTPA [8,9]. The health benefit of physical activity is proportional to its amount it [10–12] and the greatest benefit to health has been noticed with 3–5 times the WHO 2020 minimum physical activity criteria. Health benefits were also visible even when exercised abundantly (10 times the 2020 WHO minimum criteria) [13]. According to a recent systematic review, the best benefit in overall and CVD mortality from LTPA was obtained by combining resistance training with moderate-to-vigorous aerobic physical activity [14].

Although LTPA is beneficial for health, several previous studies have noticed that high occupational physical activity (OPA) can be harmful, since it could increase the risk of overall and cause-specific (such as CVD) mortality [2,5,15]. This is known as the ‘paradox of physical activitý in the literature [3]. The association between high OPA and increasing risk of overall mortality and all CVD events and CVD mortality is observed, especially in males [16,17]. It has previously been reported that a high OPA increases the incidence of all CVD events also in females [18]. A large meta-analysis made in 2021 supports the ‘paradox of physical activitý according to CVD mortality since it found a null protective effect from high OPA. Furthermore, it found a non-significant 15% increase in the risk of CVD mortality among people in physically demanding occupations [5]. However, it has been reported that after adjusting for socioeconomic status and other confounding factors, high and moderate OPA even prolonged life expectancy in males, and a similar pattern was seen in CVD mortality [17]. The knowledge of the association between LTPA and OPA is largely unknown in the literature. A systematic review conducted in 2021 found that LTPA was beneficial in lowering mortality among all degrees of physical load during working hours, but the benefit was smaller for moderate and high OPA [19]. Therefore, it is still relatively unclear whether the benefits of LTPA are seen in every OPA group, for example, in those who work heavily.

It also has to be taken into account that there are conceptual differences between OPA and LTPA. By nature, OPA is static and monotonic and often requires heavy lifting with poor ergonomy. Often OPA is too low intensity or too long duration for improving cardiorespiratory health and it is performed without sufficient recovery. On the other hand, LTPA is more controlled by intensity, frequency, duration and recovery [2,5,12]. The present study investigated the effects of LTPA and OPA on cardiovascular morbidity and mortality during a long-term follow-up. In addition, we examine how heavy work affects the benefits of leisure-time exercise.

## 2. Subjects and methods

The Oulu Project Elucidating the Risk of Atherosclerosis (OPERA) is a population-based epidemiological study focusing on the risk factors and disease endpoints of atherosclerotic cardiovascular diseases. In the first phase, the OPERA was a cross-sectional study of middle-aged (40–59 years) subjects (*n* = 1045) with hypertension (*n* = 519) and their age- and sex-matched controls (*n* = 526) [20, 21]. In our study, one study subject was rejected because of unemployment; thus, the final number of study subjects was 1044. The first phase was conducted between the years 1991 and 1993. Baseline examinations included weight, height, waist and hip measurements and blood pressure measurements. Body mass index (BMI) was calculated as weight (kg) divided by height squared (m2). A questionnaire presented to all participants provided information on their smoking habits, alcohol consumption, physical activity (LTPA and OPA), use of medication, and past medical history, including diseases. Alcohol consumption was calculated as grams of absolute alcohol consumed per week, and smoking as the number of cigarettes smoked per day [22,23]. The life-time smoking burden was calculated as pack-years (1 pack-year = 20 cigarettes smoked/day in one year), and smoking history was obtained from a questionnaire [20]. The pack years were self-reported *via* a questionnaire at the baseline of the study. We considered pack years as a predictive factor to mortality or fatal/non-fatal CVD event since we had whole study populations smoking information only at the baseline.

We used the IDF (International Diabetes Federation) definition of metabolic syndrome, and insulin sensitivity was measured using the QUICK index (quantitative insulin sensitivity check index (QUICK), as described earlier. Hypertension was defined as blood pressure (BP) > 140/90 mmHg or current antihypertensive medication [22]. The amount of liver fat was assessed using ultrasonography at baseline [24].

Routine clinical laboratory tests were performed in the Central Laboratory of Oulu University Hospital after 12-h of fasting. Hs-CRP was analyzed using commercially available ELISA kits (Diagnostic Systems Laboratories, TX, USA). Plasma cholesterol and triglyceride concentrations were determined by enzymatic colorimetric methods (Boehringer Diagnostics, Mannheim GmbH, Germany, catalog nos. 236691 and 701912), using a Kone Specific, Selective Chemistry Analyzer (Kone Instruments). Otherwise, routine clinical laboratory tests were performed in the Central Laboratory of Oulu University Hospital after 12-h of fasting as described earlier [20,21,25,26].

### 2.1. Outcome classification

The overall mortality rate was over 26 years. Information on total mortality was obtained from the Finnish Causes of Death Register. CVD mortality data were collected after the last day of 2018, with an average follow-up time of over 24 years. Fatal CV events included a major coronary heart disease (CHD) event and stroke (excluding subarachnoid haemorrhage [SAH]), whichever of them occurred first. CHD as a cause of death included I20-I25, I46, R96, R98 (ICD-10/410-414, 798 (not 7980 A) (ICD-8/9)) as an underlying cause for death or immediate causes of death and I21 or I22 (ICD-10)/410 (ICD-8/9) as first to third contributing cause of death. Stroke (excluding SAH) included I61, I63 (not I636), I64 (ICD-10)/431, 4330 A, 4331 A, 4339 A, 4340 A, 4341 A, 4349 A, 436 (ICD-9)/431 (except 43101, 43191), 433, 434, 436 (ICD-8) as main diagnosis (symptom or cause) or as first or second side diagnosis (symptom or cause) or as third side diagnosis (ICD-8/9 only) or as an underlying cause of death or immediate cause of death or as first to third contributing cause of death. Data on the occurrence of CVD and HF events were collected after the last day of 2014, with an average follow-up of over 20 years, and were obtained from the Hospital Discharge Register [27].

### 2.2. Determination of LTPA and OPA classes

We analyzed the study participants according to their LTPA and OPA. During the enrollment study, the subjects completed a health questionnaire containing questions about the frequency of habitual LTPA, smoking status, and alcohol consumption. Four LTPA groups were formed by modifying a scale originally developed by Saltin and Grimby [28]. In LTPA questionnaire OPA was excluded. The first group “no exercise” was formed by sedentary study subjects who did not exercise during leisure time except for light housework. The second group “irregular” LTPA was formed by those study subjects whose exercise included intermittent walking, cycling etc. The third group “regular” was formed by those who exercised regularly on a weekly basis 1-2 times over 30 min. The fourth group “heavy regular” was formed by those study subjects who exercised regularly over 3 times over 30 min e.g. jogging, swimming, and cycling.

When analyzing OPA, the study participants were categorized into three groups. Occupation was self-reported. Each reported occupation was manually coded to the three OPA categories by the study nurses. The first group “no activity” contained study subjects whose occupations did not include any or little activity, such as office work. The second group “mild” was formed by those whose work included mainly working while standing, moving from one place to another such as cleaner, mechanic, salesman etc. The third group “heavy” was formed by those who were in physically demanding professions such as lumberjacking, loader, etc. including heavy exercise (mainly standing, lifting, etc.) [29].

We also analyzed the effect of LTPA in different OPA groups and its effect on cardiovascular morbidity and mortality during the long-term follow-up. In this analysis, all OPA groups were divided into four different groups according to their LTPA status, making a total of 12 groups. In some groups, especially in the sedentary OPA groups (“no exercise”), the group sizes were small. Especially in the “heavy” OPA groups, the lack of women played a role in the analyses; thus, we also analyzed females and males separately when considering the effect of LTPA on OPA groups.

### 2.3. Statistical methods

Data were analyzed using IBM SPSS Statistics version 27. Analysis of variance (ANOVA) was used to compare LTPA and OPA groups among themselves with continuous variables. Post-hoc analyses of differences between groups were conducted using Tukeýs statistical test. The chi-squared test (*χ^2^*) was used for categorical variables. Statistical significance was set at *p* < 0.05.

Total and cardiovascular mortality was assessed as the cumulative proportional probability of development of deaths and CVD events and analyzed using Kaplan-Meier survival curves for physical activity groups. The statistical significance of the Kaplan-Meier survival curves was calculated using the log-rank test. Kaplan Meier survival curves were created using the R Core Team (2021). R: A language and environment for statistical computing. R Foundation for Statistical Computing, Vienna, Austria. URL https://www.R-project.org/. The secondary survival analysis, taking into account confounding factors, was performed with Cox proportional hazards models. We used age, sex, smoking pack years, HTA, LDL cholesterol, BMI, and alcohol consumption as confounding factors. As a reference group for LTPA, we used the most physically active group (“heavy”) and in OPA the most sedentary group (“no activity”).

### 2.4. Ethical considerations

This study was approved by the Ethics Committee of the Medical Department of the University of Oulu (48/2009). Written informed consent was obtained from all participants for the use of their clinical records.

## 3. Results

### 3.1. Clinical characteristics

#### 3.1.1. Leisure time activity

Females were over-represented, especially in the “no exercise” group (Table 1), and 70.3% of the participants were females (*p* = 0.001). In other groups, the sex distribution was more even. The study subjects in the “heavy regular” group were the oldest. The age of the “heavy regular” group was higher compared with the “irregular” and “regular” groups. (*p* < 0.001)

**Table 1.** Clinical characteristics, leisure time activity.

	1 No exercise	2 Irregular	3 Regular	4 Heavy regular	*p* value	Post hoc
	*n* = 64	*n* = 302	*n* = 329	*n* = 349		
Age (years)	51.1	50.5	50.4	52.8	<0.001	e,f***
Female (%)	70.3	44.7	47.7	53.9	0.001	
BMI	30.4	28.7	27.3	26.7	<0.001	a*,b,c,e***, d**
Height (cm)	164.8	168.5	169.0	167.0	0.001	a,f*,b**
Weight (kg)	82.9	81.8	78.4	74.5	<0.001	c,e***,d*, f**
Waist circumference (cm)	96.4	93.6	90.3	87.3	<0.001	b,c**,d,e***,f*
Smoking pack years (years)	15.3	12.1	9.0	7.7	<0.001	c,e***,d*
Alcohol consumption (g/week)	86.7	72.5	60.1	47.2	<0.001	c,e**

BMI: body mass index. Data for continuous variables are tested with ANOVA. Categorical variables are as prevalence (%) and tested with Pearson Chi-square test. For post hoc analyses, *p < 0.05, **p < 0.01, ***p < 0.001, analyzed using ANOVA Tukey statistical test (95 % confidence interval) for differences between groups (a) 1 vs 2; (b) 1 vs 3 (c) 1 vs 4; (d) 2 vs 3; € 2 vs 4; (f) 3 vs 4.

Due to females’ over-representation in the “no exercise” group, differences in the height of the study subjects existed. In the “no exercise” group the mean height was the shortest, while in the “regular” group, the mean height was the highest (*p* = 0.001). When considering weight, physical activity-related differences were observed between groups. The mean weight decreased when the activity of the study participants increased. The mean weight of the “heavy regular” group was lowest, while it was highest in the “no exercise” group (*p* < 0.001). The same trend was observed for mean waist circumference (*p* < 0.001). The differences in weight previewed the differences in BMI. Accordingly, an activity-related decrease in mean BMI occurred between the groups. BMI was lowest in the “heavy regular” group and highest in the “no exercise” group (*p* < 0.001).

In addition, the use of tobacco products and alcohol was reflected in activity trends between groups. The study participants who exercised less used more alcohol (*p* < 0.001) and tobacco products (*p* < 0.001). The “heavy regular” group averaged half of the pack years and alcohol consumption (gr/week) than the “no exercise” group.

When considering morbidities (Table 2), statistically significant differences between groups were observed in the incidence of HTA, MBO, and fatty liver disease. The study subjects who exercised more were in a more favourable situation considering metabolic abnormalities. “Regular” and “heavy regular” groups had the lowest incidence rate and the “no exercise” group had the highest incidence rate of HTA (*p* < 0.001) at the beginning of the study. When considering MBO, the incidence decreased as physical activity increased (*p* < 0.001). In the “no exercise” and “irregular” exercise groups, there was more moderate to severe liver brightness than in the “regular” and “heavy regular” groups. (*p* < 0.001)

**Table 2.** Morbidities, leisure time activity.

	No exercise	Irregular	Regular	Heavy regular	*p* value
	*n* = 64	*n* = 302	*n* = 329	*n* = 349	
HTA (%)	75.0	57.6	47.7	45.8	<0.001
DM (%)	12.5	11.3	7.9	10.9	0.677
MBO (%)	56.3	48.7	35.6	25.2	<0.001
Use of HC drug (%)	0	2.6	2.1	4.0	0.095
MCC (%)	11.3	5.2	8.6	10.7	0.105
AMI (%)	1.6	1.0	2.4	2.3	0.183
Stroke (%)	7.8	2.0	0.6	2.6	0.334
IMT (cm)	0.84	0.88	0.87	0.88	0.460
Kidney disease (%)	6.3	3.3	3.0	4.0	0.691
Fatty liver (%)	35.5	35.7	25.6	20.3	<0.001
Lung disease (%)	9.4	9.9	9.1	9.2	0.784
Thyroid disease (%)	7.8	6.0	7.6	8.3	0.374

HTA: hypertension; DM: diabetes; MBO: metabolic syndrome; HC: hypercholesterolemia; MCC: coronary heart disease; AMI: acute myocardial infarction, IMT: intima media thickness. Categorical variables are as prevalence (%) and tested with Pearson Chi-square test.

Hemoglobin was the highest in the “irregular” group (*p* < 0.001) and the difference was statistically significant compared to “no exercise” group and the “heavy regular” group (Table 3). High-sensitivity C-reactive protein (hsCRP) was the highest in the “no exercise” group (*p* < 0.001). Triglycerides were the lowest (*p* < 0.001), while HDL cholesterol was the highest in the “heavy regular” group (*p* = 0.001). However, the difference in HDL cholesterol levels was statistically significant only between the “heavy regular” and “irregular” groups.

**Table 3.** Laboratory measurements, leisure time activity.

	1 No exercise	2 Irregular	3 Regular	4 Heavy regular	*p* value	Post hoc
	*n* = 64	*n* = 302	*n* = 329	*n* = 349		
Hb (g/l)	138	145	143	140	<0.001	a**,e***
hsCRP (mg/mL)	8.3	4.2	2.9	3,5	<0.001	a,b,c***
ALAT (U/l)	35	33	33	30	0.110	
Creatinine (µmol/l)	92	83	82	82	0.096	
GFR (CKD-EPI) mL/min	83	85	85	83	0.093	
Cholesterol (mmol/l))	5.71	5.71	5.71	5.69	0.992	
Triglyserides (mmol/l)	1.92	1.75	1.58	1.38	<0.001	c,**e***,f*
HDL cholesterol (mmol/l))	1.28	1.30	1.34	1.41	0.001	e**
LDL cholesterol (mmol/l)	3.51	3.53	3.54	3.51	0.978	

Hb: hemoglobin; hsCRP: high-sensitivity C-reactive protein; ALAT: alanine aminotransferase; GFR (CKD-EPI): estimated glomerular filtration calculated with CKD-EPI formula (Chronic Kidney Disease Epidemiologic Collaboration), HDL cholesterol, high density lipoprotein, LDL cholesterol, low density lipoprotein. Data for continuous variables are tested with ANOVA. For post hoc analyses, *p < 0.05, **p < 0.01, ***p < 0.001, analyzed using ANOVA Tukey statistical test (95 % confidence interval) for differences between groups (a) 1 vs 2; (b) 1 vs 3 (c) 1 vs 4; (d) 2 vs 3; (e) 2 vs 4; (f) 3 vs 4.

#### 3.1.2. Occupational activity

The “heavy” group was formed almost entirely by males, females represented only 6,8% of this group (*p* < 0.001)(Table 4). On the other hand, the gender distribution was almost even in the “no activity” group and “mild” group. The “mild” group was the oldest (*p* = 0.001).

**Table 4.** Clinical characteristics, occupational activity.

	1 No activity	2 Mild	3 Heavy	*p* value	Post hoc
	*n* = 427	*n* = 544	*n* = 73		
Age (yrs)	50.7	51.9	50.0	0.001	a,c*
Female (%)	222 (52.0)	298 (54.8)	5 (6.8)	<0.001	
BMI	27.7	27.7	27.8	0.970	
Height (cm)	169.0	166.3	173.4	<0.001	a,c***,b**
Weight (kg)	79.5	76.8	83.8	<0.001	a*,c**
Waist circumference(cm)	90.7	89.8	95.8	0.001	b,c**
Smoking pack years (years)	8.7	10.3	13.4	0.024	b*
Alcohol consumption (g/week)	65.7	54.6	81.2	0.022	c*

BMI: body mass index. Data for continuous variables are tested with ANOVA. Categorical variables are as prevalence (%) and tested with Pearson Chi-square test. For post hoc analyses, *p < 0.05, **p < 0.01, ***p < 0.001, analyzed using ANOVA Tukey statistical test (95 % confidence interval) for differences between groups (a) 1 vs 2; (b) 1 vs 3; (c) 2 vs 3.

The “heavy” group (mostly male) was the tallest group by average height and the “mild” on the other hand was the shortest (*p* < 0.001). The average weight in the “mild” group was lower than that in the other groups (*p* < 0.001). Waist circumference was the largest in the “heavy” group compared to other groups (*p* = 0.001). However, despite the differences in weight and height, there were no statistically significant differences in BMI between the groups (*p* = 0.970).

In the “heavy” group study subjects had smoked the most pack years (*p* = 0.024) while the “no activity” group had smoked the least pack-years. The “heavy” group also used alcohol the most while the “mild” group the least at the baseline (*p* = 0.022). Statistically significant differences between morbidities at the beginning of the study were not evident.

Haemoglobin and eGFR were highest in the “heavy” group (*p* < 0.001)(Table 5 and supplement Table). Also, the significant difference between the “heavy” group and “mild” group occurred in HDL cholesterol (*p* = 0.041, according to post hoc analysis but p for trend was not significant) It seemed that the “heavy” group had the highest LDL cholesterol level (*p* = 0.035), however, post hoc analysis did not reveal any significant differences between groups.

**Table 5.** Laboratory measurements, occupational activity.

	1 No activity	2 Mild	3 Heavy	*p* value	Post hoc
	*n* = 427	*n* = 544	*n* = 73		
Hb (g/l)	142	141	150	**<0.001**	b,c,***
hsCRP (mg/mL)	3.8	3.6	4.8	0.488	
ALAT (U/l)	34	31	33	0.125	
Creatinine (µmol/l)	82	83	83	0.911	
GFR (CKD-EPI) mL/min	84	84	92	**<0.001**	b,c***
Cholesterol (mmol/l))	5.61	5.76	5.75	0.254	
Triglyserides (mmol/l)	1.61	1.55	1.66	0.076	
HDL cholesterol (mmol/l))	1.35	1.36	1.25	0.058	c*
LDL cholesterol (mmol/l)	3.44	3.58	3.67	**0.035**	

Hb: hemoglobin; hsCRP: high-sensitivity C-reactive protein; ALAT: alanine aminotransferase; GFR (CKD-EPI): estimated glomerular filtration calculated with CKD-EPI formula (Chronic Kidney Disease Epidemiologic Collaboration), HDL cholesterol: high density lipoprotein, LDL cholesterol: low density lipoprotein. Data for continuous variables are tested with ANOVA. For post hoc analyses, *p < 0.05, **p < 0.01, ***p < 0.001, analyzed using ANOVA Tukey statistical test (95 % confidence interval) for differences between groups (a) 1 vs 2; (b) 1 vs 3; (c) 2 vs 3.

### 3.2. Survival

#### 3.2.1. Leisure time activity

There were no significant differences in the total mortality between the groups. In addition, there were no significant differences between the groups in terms of cardiovascular events and deaths caused by cardiovascular events. There were also no statistically significant differences between groups according to adjustments (age, smoking pack years, HTA, LDL cholesterol, BMI, and alcohol consumption) when sex was analyzed separately.

**Figure 1.**
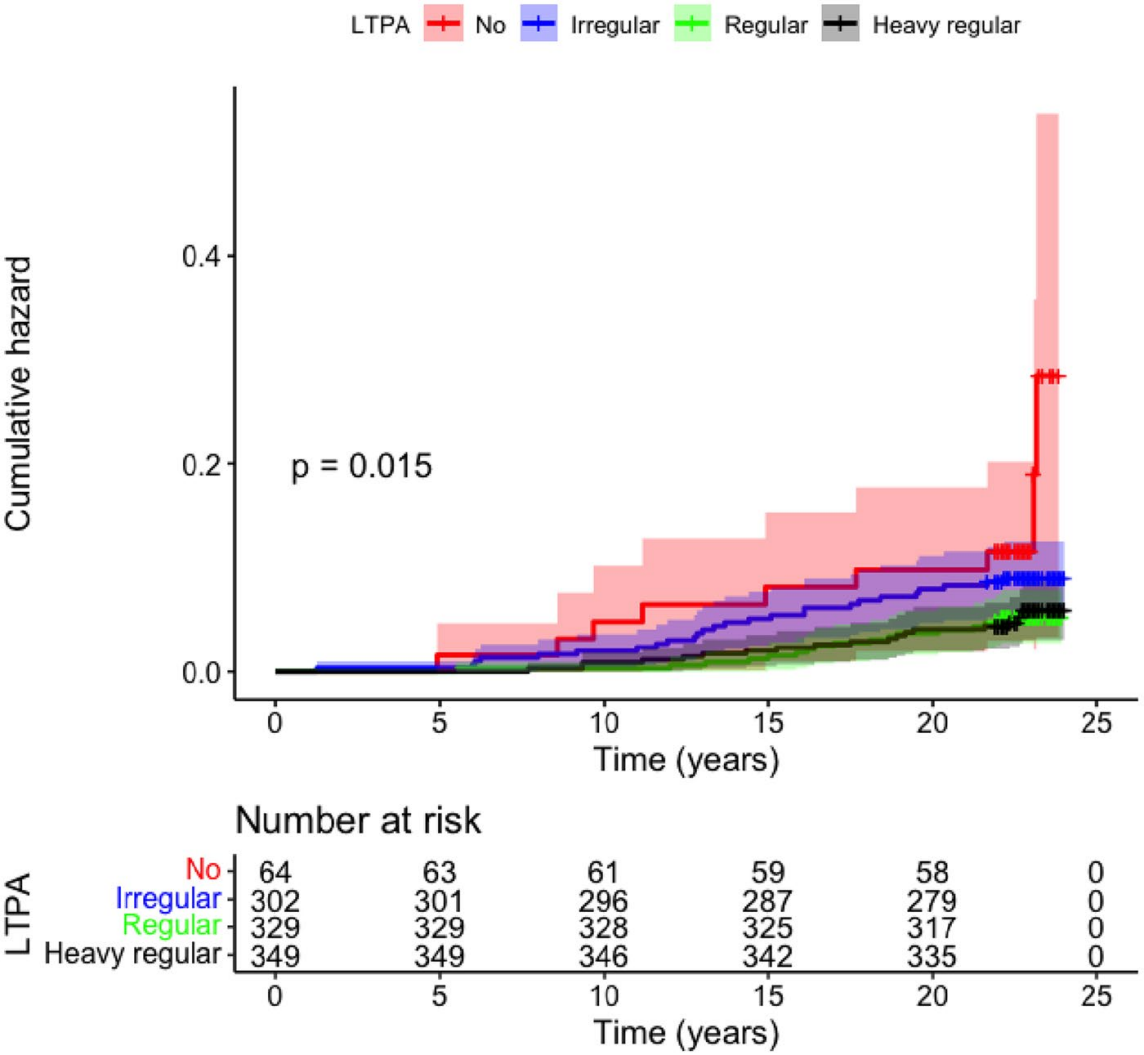
Kaplan–Meier cumulative hazard analysis for heart failure among leisure-time physical activity groups.

However, in the “no exercise” group the occurrence of heart failure was increased compared to other groups (*p* = 0.015) (Figure 1). After adjustments in the Cox regression analysis (Table 6), the differences in the heart failure rate between the groups were visible. In the “no exercise” group, the HR of heart failure was 2.585 (CI 95% 1.107 − 6.036). 14.1% of the “no exercise” group developed heart failure while only 5.1-8.6% in the other groups developed heart failure. Analysing genders separately There were no statistically significant differences between the groups after adjusting for age, smoking pack years, HTA, LDL cholesterol, BMI, and alcohol consumption.

**Table 6.** Heart failure at the end of 2014, leisure time activity, ”heavy regular” group as a reference.

	Total mortality	Number of events
	HR (95% CI)	(%)
Exercise status group		
Heavy regular (*n* = 349)	1	18 (5.2)
No exercise (*n* = 64)	2.585 (1.107; 6.036)	9 (14.1)
Irregular (*n* = 302)	1.714 (0.914; 3.215)	26 (8.6)
Regular (*n* = 329)	1.162 (0.592; 2.279	17 (5.1)
Sex (male)	1.486 (0.839; 2.633)	
Age (years)	1.105 (1.056; 1.156)	
Smoking (pack years)	1.011 (0.997; 1.024)	
HTA	1.319 (0.789; 2.204)	
LDL cholesterol (mmol/l)	0.952 (0.737; 1.229)	
BMI (kg/m^2^)	1.063 (1.012; 1.116)	
Alcohol (g/wk)	1.002 (1.000; 1.004)	

#### 3.2.2. Occupational activity

**Figure 2.**
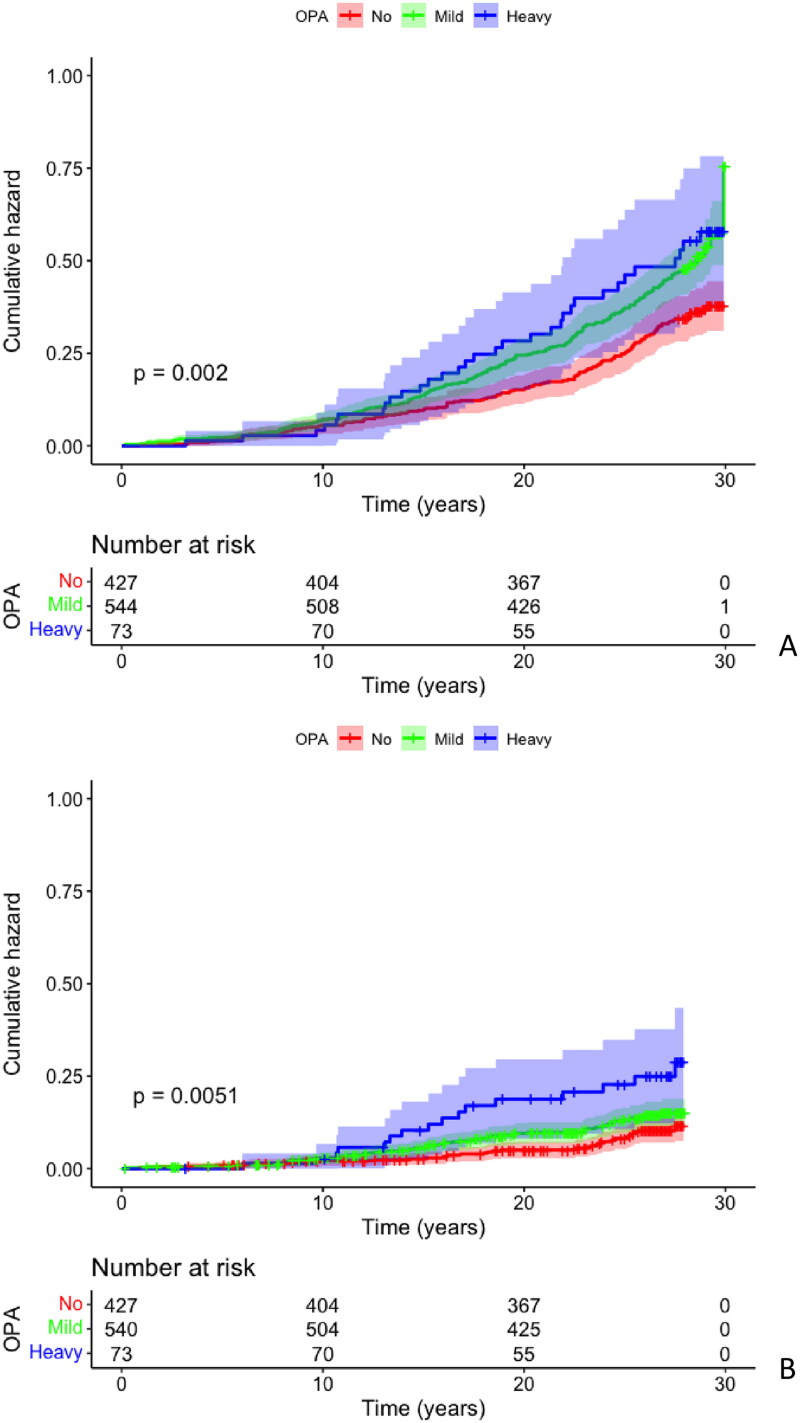
Kaplan–Meier cumulative hazard analysis for overall mortality (A) and fatal cardiovascular disease events (B) among occupational physical activity groups.

When considering working time physical activity total mortality (between the years 1993 and 2020) was increased in “heavy” and “mild” groups according to Kaplan–Meier analysis (*p* = 0.002) (Figure 2(A)). Table 7 shows that after adjustments only in the “mild” group, the total mortality was significantly increased with an HR of 1.384 (CI 95% 1.112 − 1.721). In the “heavy” group, where the overall number of study subjects is much smaller, the HR of total mortality was only slightly lower (1.324) than in the “mild” group, but the CI95% was from 0.894 to 1.960 (Table 7), even though the percentage of events was slightly higher than in the “mild” group. Analyzing genders separately, the same result was seen only in males. The risk of total mortality was statistically significantly increased after adjustments in the “mild” group compared to the “no activity” group, the HR of total mortality was 1.567 (CI 95% 1.179 − 2.083).

**Table 7.** Overall mortality at the end of 2020, occupational activity, “no activity” group as a reference.

	Total mortality	Number of events
	HR (95% CI)	(%)
Exercise status group		
No activity *(n* =427)	1	131 (30.7)
Mild (*n* = 544)	1.384 (1.112; 1.721)	222 (40.8)
Heavy (*n* = 73)	1.324 (0.894; 1.960)	32 (43.8)
Sex (male)	1.404 (1.106; 1.781)	
Age (years)	1.105 (1.084; 1.126)	
Smoking (pack years)	1.021 (1.015; 1.126)	
HTA	1.179 (0.955; 1.445)	
LDL cholesterol (mmol/l)	1.013 (0.906; 1.132)	
BMI (kg/m^2^)	1.024 (1.001; 1.047)	
Alcohol (g/wk)	1.002 (1.001; 1.003)	

The incidence of all cardiovascular events was increased especially in the “heavy” group (*p* = 0.004). 38.5% of the “heavy” group had some CVD event, while the same percentage was 20.4-25.6% in the other groups. However, after adjustments, there were no statistically significant differences in cardiovascular events between the groups. Figure 2 (B) shows that especially in the “heavy” group, the mortality caused by cardiovascular events increased (*p* = 0.005). The percentage of CVD-related deaths was 21.9, while in the other groups, it was only 9.1–12.1. Table 8 shows that after adjustment, the risk of CVD mortality increased in the “heavy” group. The HR for death caused by CVD events was 1.943 and the CI 95% was statistically significant (1.077 − 3.503). There were no statistically significant differences between the groups after adjustments for fatal and non-fatal CVD events when analyzing sex separately.

**Table 8.** CVD mortality at the end of 2018, occupational activity, “no activity” group as a reference.

	Total mortality	Number of events
	HR (95% CI)	(%)
Exercise status group		
No activity (*n* = 427)	1	39 (9.1)
Mild (*n* = 544)	1.401 (0.938; 2.092)	66 (12.1)
Heavy (*n* = 73)	1.943 (1.077; 3.503)	16 (21.9)
Sex (male)	2.100 (1.327; 3.323)	
Age (years)	1.104 (1.067; 1.141)	
Smoking (pack years)	1.025 (1.015; 1.035)	
HTA	1.218 (0.832; 1.783)	
LDL cholesterol (mmol/l)	1.157 (0.953; 1.404)	
BMI (kg/m^2^)	1.043 (1.003; 1.085)	
Alcohol (g/wk)	1.002 (1.000; 1.003)	

There was no significant difference between the groups in terms of the risk of heart failure in different OPA groups (*p* = 0.632). There were also no statistically significant differences between groups when analyzing sex separately.

When LTPA was added to the list of confounding factors it did not change significantly the original results for OPA in the case of overall mortality, fatal and non-fatal CVD events and HF. Vise versa, when analysing LTPA and OPA was added to the list of confounding factors we did not notice any significant change in the results.

#### 3.2.3. Survival analysis of OPA in different LTPA groups

The total mortality did not differ when the OPA groups were analyzed according to their activity during leisure time (data not shown). However, after splitting the OPA groups by LTPA, the number of subjects in the different groups was too small to make relevant comparisons.

#### 3.2.4. Survival analysis of LTPA in different OPA groups

**Figure 3.**
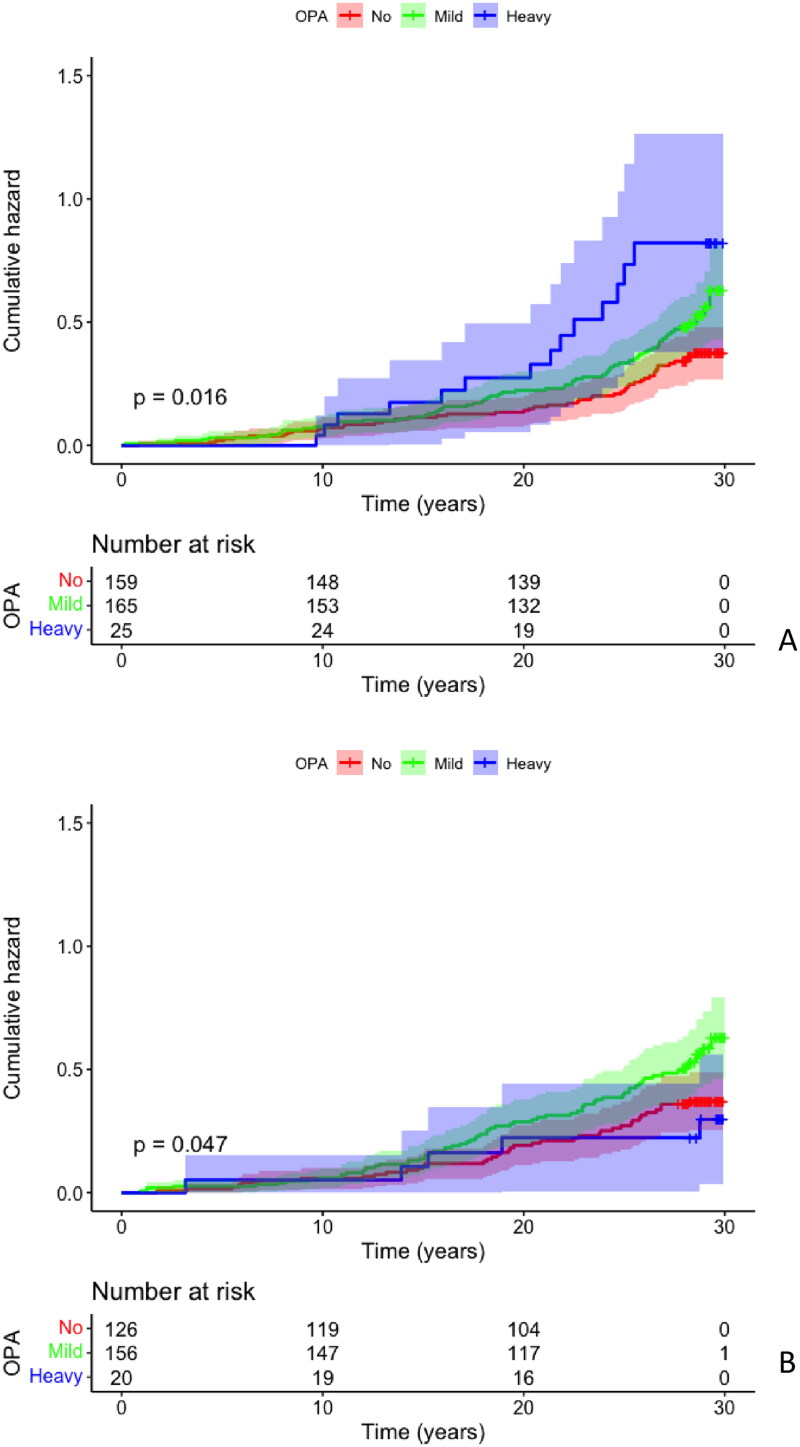
Kaplan–Meier cumulative hazard analysis for overall mortality among different occupational physical activity groups. The analyses were performed separately based on patients’ leisure-time physical activity. Panel A: “heavy regular” leisure-time physical activity. Panel B: “irregular” leisure-time physical activity.

There were no statistically significant differences in the LTPA habits between the OPA groups (*p* = 0.187). This implies that all the workers were physically active during their leisure time. When LTPA groups were analyzed according to their occupational activity, before adjustments, “heavy regular” LTPA was associated with the highest mortality among subjects in demanding occupations (“heavy” OPA). The overall survival rate was the lowest at 44%, while the same percentage in the “mild” OPA and “heavy regular” LTPA group was 59% and in the “no activity” OPA and “heavy regular” LTPA groups was 69%. Figure 3 A shows the cumulative hazard ratio for overall mortality (*p* = 0.016). The HR in the “heavy” group increased significantly compared to the “no exercise” group (*p* = 0.008, CI 95%; 2.224; 1.226–4.033). However, after adjustment, this finding was not significant (*p* = 0.136). Due to the lack of women in the “heavy” OPA groups, the statistically significant difference after adjusting for sex disappeared. Thus, we analyzed the sexes separately to examine the differences between males. However, there were also no statistically significant differences between the groups when analyzing only males (*p* = 0.139).

In the “irregular” LTPA group, there were significant differences before and after adjustments in the hazard ratios in different OPA groups (Figure 3(B)). When LTPA was “irregular”, the “mild” OPA group subjects experienced an overall survival rate of 56%, while the same percentage in the “no activity” OPA group was 69% and the survival was the highest in the “heavy” OPA group 75% (*p* = 0.047) When comparing the “mild” group and the “no activity” group the HR of overall mortality in the “mild” group was significantly increased (*p* = 0.032, CI95%; 1.539; 1.038–2.282). This finding was also statistically significant after adjustments were made with Cox regression analysis (*p* = 0.041, CI95%; 1.514; 1.017–2.254) and remained statistically significant when only males were considered in the analysis (*p* = 0.007, CI95% 2.041; 1.216-3.425). There were no significant differences in total mortality in the other OPA groups when considering LTPA (*p* = 0.682 and *p* = 0.146).

When considering CVD events over 20 years follow, we noticed that there were statistically significant differences in the OPA groups who had “heavy regular” activity in their leisure time. In the latter LTPA group, the occurrence of being free of fatal and non-fatal CVD events in the “heavy” OPA group was lower especially compared to “no activity” OPA group (*p* = 0.006). In the other LTPA groups, there were no statistically significant differences in the CVD events between the OPA groups. After adjusting for confounding factors (sex, age, smoking pack-years, HTA, LDL cholesterol, BMI, and alcohol consumption), these associations were lost (*p* = 0.160). There were also no statistically significant differences when analyzing males separately (*p* = 0.328). When sex was removed from the adjustment, the difference was statistically significant. This finding implies that the difference was caused by the gender effect.

As mentioned above, considering fatal CVD events in 24 year follow-up in different LTPA groups according to their OPA group, we noticed by Kaplan-Meier analysis that “regular” or “heavy regular” LTPA was harmful for study subjects who were in physically demanding occupations (“heavy”). In the “heavy regular” or “regular” LTPA groups occurrence of being free of fatal CVD events was lower in the “heavy” OPA group than in the other OPA groups. (*p* = 0.007 and 0.021, respectively). In the other LTPA groups, we did not observe any significant differences between the OPA groups. After adjusting (sex, age, smoking pack years, HTA, LDL cholesterol, BMI, and alcohol consumption) there were no statistically significant differences between the groups (*p* = 0.104). Like above in all CVD events, also in fatal CVD events it seemed that the difference was formed between males and females, since when sex was removed from adjustments, the difference became statistically significant. However, after analyzing males separately, there was no statistically significant difference between the groups (*p* = 0.181). There were no statistically significant differences in the incidence of HF between the LTPA and OPA groups.

## 4. Discussion

The present study shows that regular LTPA protects study subjects from heart failure but not from other cardiovascular events in a 20-year follow-up period. Overall mortality at 26-year follow-up was higher in the “mild” OPA compared to “no activity” group. CVD mortality was significantly higher in the” heavy” OPA compared to “no activity” group. “Heavy” OPA study subjects benefitted from “irregular” but not “regular” or “heavy regular” LTPA.

Previous studies have shown that overall physical activity reduces the risk of overall mortality [3,5,6]. It has been reported that leisure-time physical (aerobic and muscle strengthening) activity has a significantly lowering impact on overall mortality, with a nearly 40% reduction effect, compared to inactive people. When considered separately, aerobic activities may reduce the risk of total mortality by nearly 29% and muscle-strengthening activities by 11% compared with inactive people [30]. A meta-analysis conducted in 2019 reported that physical activity had an inverse relationship with overall survival [31]. Study subjects who were moderately to vigorously physically active had a reduced risk of overall mortality. Somewhat inconsistent with previous studies, LTPA did not predict total or cardiovascular mortality in our study. It has to be kept in mind, however, that in our “heavy regular” group the minimum amount of LTPA was 3 times at least 30 min workout which adds up to only 90 min of active training per week. The WHO recommends at least 150-300 min per week of moderate-intensity or 75–150 min of vigorous-intensity physical activity or some equivalent combination of moderate/vigorous-intensity physical activity [8]. This may partly explain why LTPA and especially higher doses of LTPA seemed not to provide health benefits. The number of subjects in the main reference group (”no exercise”) was relatively small. Women were also over-represented in the main reference group, and it has been noted that the incidence of CVD events in women is lower than that in men [32]. On the other hand, previous studies have noticed that even 1–2 times per week moderate/vigorous physical activity during leisure time lowers the risk of CVD events. Although the 1–2 sessions in the “weekend warriors” were long enough to cover up the WHO minimum recommended time for physical activity (150–300 min). On the other hand in our study, the “regular” (who exercised also 1–2 times per week) exercised significantly less (60–90 min) compared to the “weekend warriors.” This might explain partly why the “regular” (and less “heavy regular”) active group didn’t show any statistically significant benefits on results on overall mortality and fatal and non-fatal CVD events [6].

Importantly, the effects of leisure time and occupational physical activity on survival may be different, and the majority of earlier studies have not separated physical activity based on occupational or leisure time activities [31]. Supporting the latter notion, in a 10-year follow-up, heavy OPA increased significantly, while regular LTPA reduced total mortality. This effect is called the paradox of physical activity [3]. The increased risk of mortality among heavy OPA is noticed, especially in males [33,34]. Similarly to these observations, our study subjects belonging to the “mild” or “heavy” OPA groups experienced a higher risk of total mortality compared to those who did not have any physical activity at work. After adjusting for confounding factors, only the “mild” group showed higher risk than “no activity” group. This was also seen in males when analyzing sex separately. The small number of subjects (*n* = 73) in the “heavy” group might partly explain why the HR was not significantly increased compared to the “no activity” group. Also due to the males over-representation, the “heavy” group in females got small when analyzing genders separately. People with more sedentary jobs exercise more during their leisure time. A systematic review conducted in 2021 found that LTPA in general is beneficial, but its positive impact is significantly lower in subjects in physically demanding occupations [19]. However, in our study, we did not notice any differences in the study subjects’ LTPA according to their OPA group. It has to be taken into consideration that the number of subjects was relatively small when the OPA group was divided according to LTPA. Our study results are more or less consistent with the findings of Prince et al. 2021 [19]. Although the positive impact of regular LTPA was lost, it was harmful in subjects who were in physically demanding occupations. Our findings support the notion that subjects who exercise heavily during their workday should not exercise regularly or too heavily during their leisure time. Meta-analysis made in 2018 highlighted the fact that OPA itself could be the factor causing increased mortality among “heavy” workers. They speculated that people should have different physical activity recommendations based on their OPA status [15]. In our study, the best survival benefit among the “heavy “workers was in the “irregular” LTPA. Since the number of study subjects in the “no exercise” LTPA group was too small to make any relevant conclusions, we can not assess whether high OPA workers benefit from even less exercise than the “irregular”. However, it is still visible according to our study that high OPA workers benefit from less leisure time exercise than WHO PA guidelines recommend. High OPA, in general, consists mainly of musculoskeletal strength required by static and monotonic movements combined with a long duration without sufficient recovery times [2,5,15]. Depending on occupation and the intensity and duration related to it high OPA subjects fulfil current physical activity recommendations from a time and workload point of view. In some cases, they might exceed the recommendations even several ten times. From a cardiorespiratory fitness point of view, a high dose of OPA is of too low intensity or too long duration and it might not meet the PA recommendations [2]. Taking into account the above, it is not necessarily recommendable to target LTPA goals with high-intensity aerobic or resistance training, especially in the amount that WHO currently recommends. Instead, we suggest high OPA subjects from an intensity and duration point of view to focus on LTPA that supports or even improves recovery from physically demanding occupations. From a cardiorespiratory point of view, for example, low-intensity cycling, jogging, swimming or walking could be suitable. In turn from a resistance training point of view, physical therapy like training which improves recovery could be suitable. On the other hand, both cardiorespiratory fitness and musculoskeletal strength-improving exercise is recommendable to more sedentary workers [14]. However, recent studies have noted that high OPA does not necessarily increase the overall risk of mortality [35]. It has been noticed that after adjustments for socioeconomic status and other confounding factors, high and moderate OPA could even prolong life expectancy, while with no adjustments, the overall mortality and CVD mortality increased in higher OPA groups [17,36].

In our study, neither OPA nor LTPA predicted non-fatal CVD events during the 20-year follow-up period. A meta-analysis conducted in 2019 observed an inverse relationship between overall physical activity and non-fatal and fatal CVD events [31]. For cardiovascular mortality, a systematic review conducted in 2021 found that LTPA was similarly beneficial in all OPA groups [19]. However, LTPA has been reported to reduce the risk of cardiovascular events, while OPA increases them [3]. In addition, a recent meta-analysis did not observe a beneficial effect of OPA on CVD mortality, but a non-significant 15% increased risk among workers with higher levels of physical activity was observed. Our findings in this study support the results of Cillekens et al. [5], showing a statistically significant increase in the risk of death caused by CVD events in the “heavy” workers. According to these studies, it seems that a high amount of occupational physical activity or unknown factors associated with it could be unfavourable for cardiovascular health. OPA increasing the risk of fatal and non-fatal CVD events has been noticed previously only in males. However, it has been noted that high OPA could increase the risk of all CVD events in females also [17,18]. In our study, we did not notice any difference in the 20–24 year follow-up in the non-fatal and fatal CVD events when analyzing males. In females, the small N in the “heavy” group challenges the analysis, however, there was no significant difference between “no activity” and “mild” groups. A similar finding was observed in a recent study with Mediterranean people [36]. However, a recent study with 34-year follow-up noticed that high OPA increased the risk of all CVD events in males, but could even lower the risk in females [16].

Our study showed that LTPA but not OPA predicted the risk of HF. Study subjects who did not exercise (“no-exercise”) at their leisure time had a higher risk to develop heart failure than those who exercised regularly over 30 min in 3 days per week (“heavy regular”). Our findings highlight the importance of LTPA in heart function preservation. Even though the incidence of CVD events seemed to be increased in the “heavy” OPA group the risk of heart failure was not increased. HF is still a moderately frequent complication of myocardial infarction despite advanced treatments in the past decades [37]. Previous studies have reported that endurance training improves left ventricular function and overall cardiovascular fitness [38]. Studies have noted that a higher amount of overall physical activity or physical activity separated by LTPA and OPA reduces the risk of heart failure. Moreover, overall cardiovascular fitness is associated with a reduced risk of heart failure [39]. Combining different types of physical activity increases the risk of heart failure [40]. This finding is seen in our study, even though the overall LTPA even in the “heavy regular” group was relatively small compared to WHO recommendations.

In conclusion, LTPA seems to protect middle-aged study participants from heart failure in the long term. Overall mortality in 26-year follow-up was increased in the “mild” and CVD mortality in the” heavy” OPA group compared to “no activity” OPA. It seemed that study participants who were in physically demanding occupations benefitted more from less LTPA than currently recommended by the WHO. Thus, we suggest targeting different LTPA recommendations to different OPA groups.

## Strengths and limitations

A main strength of this study is its long follow-up times according to mortality and morbidities. The routine test measured with study subjects made it possible to analyse the results taking confounding factors into account broadly. Also, further analysis and follow-up studies about this subject are possible to make from this OPERA-based study. As mentioned earlier the recent physical activity guidelines do not separate the quality of physical activity. In this context also literature has not yet studied broadly the association of LTPA and OPA.

Even though the study population was sufficient, it is not comprehensive enough to make reliable population-level conclusions. Our study population is relatively small, although more precise, than other studies in this subject. The relatively small study population made some analyses difficult to make, especially when the number of interpretable subgroups increased (for example analysing OPA according to their LTPA). Recent studies have found that the increase in mortality is not caused by OPA itself, but is more associated with socioeconomic status [17,36]. However, in our study, we had no further information about the study subjects’ socioeconomic status. In future studies, socioeconomic status and the factors behind it should be taken into consideration.

When analysing mortality and morbidity according to the study subject’s LTPA, we came across a problem the amount of LTPA was quite low in every LTPA group. Even in the “heavy regular” group the minimum amount of exercise is lower compared to what WHO recommends. However, in the “heavy regular” group there were subjects who exceeded WHO recommendations clearly. In addition, we were not able to analyse the intensity and duration of the different kinds of intensity precisely of LTPA in study subjects. Currently WHO have different recommendations for physical activity based on intensity. Moderate to vigorous intensity physical activity requires only half the time than low-intensity activity. Due to a lack of proper intensity information, we cannot be certain if participants meet or don’t meet guidelines.

The amount of OPA and LTPA was assessed at the baseline of the study from self-reported questionary and the group formation based on PA was made by that time point. This means that the study does not take into account the possible changes in physical activity habits and can not analyse the dose dependency of LTPA and OPA in relation to mortality and morbidities. The stability of the responses to the physical activity questions over time remains also unclear. Also, a major part of the study population retired from their occupation during follow-up. Furthermore, the study does not separate the quantity of LTPA, and it does not further take into account the type of occupation or physical activity in it. This leaves some occupation-related confounding factors unanalysed. For example, the different types of strain caused by work and some occupation-related other factors like pollutants, air quality or inadequate recovery caused by shift work.

## Data Availability

The data that support the findings of this study are available upon request from the corresponding author [OU]. The data were not publicly available because of restrictions containing information that could compromise the privacy of the research participants.
